# Multiplexed on-site sample-in-result-out test through microfluidic real-time PCR (MONITOR) for the detection of multiple pathogens causing influenza-like illness

**DOI:** 10.1128/spectrum.02320-23

**Published:** 2023-10-27

**Authors:** Yi Yang, Chao Wang, Hua Shi, Xudong Guo, Wanying Liu, Jinhui Li, Lizhong Li, Jun Zhao, Guohao Zhang, Hongbin Song, Rongzhang Hao, Rongtao Zhao

**Affiliations:** 1 Chinese PLA Center for Disease Control and Prevention, Beijing, China; 2 Beijing Baicare Biotechnology Co., Ltd., Beijing, China; 3 Department of Toxicology and Sanitary Chemistry, School of Public Health, Capital Medical University, Beijing, China; Beijing Institute of Microbiology and Epidemiology, Beijing, China

**Keywords:** influenza-like illness, microfluidic technology, multi-pathogen detection, SARS-CoV-2, influenza A virus, monkeypox virus

## Abstract

The global COVID-19 pandemic and frequent outbreaks of other infectious diseases, such as influenza and monkeypox, often initially manifest with non-specific influenza-like symptoms. Portability to the outbreak site and accurate identification of the various pathogens causing influenza-like illness are crucial for the rapid implementation of effective control measures. Utilizing real-time PCR and microfluidic technology, a multiplexed on-site sample-in-result-out test through microfluidic real-time PCR (MONITOR) has been developed for detecting the pathogens responsible for influenza-like illness. The operator simply needs to introduce the sample to a highly integrated microfluidic chip (requiring ≤1 minute), and the system can autonomously execute sample pre-processing, nucleic acid extraction, and PCR amplification and deliver results for eight pathogens within 85 minutes. The detection limit of MONITOR for the eight pathogens ranges from 0.78 to 6.25 copies/µL. Standard curves demonstrate notable linearity and amplification efficiency. A simulated clinical sample test reveals MONITOR’s sensitivity, specificity, and accuracy at 97.5%, 100%, and 98%, respectively. Bland-Altman analysis demonstrates strong agreement between the cycle threshold of positive MONITOR samples and quantitative polymerase chain reaction (qPCR) (*R* ^2^ = 0.952), suggesting MONITOR’s ability to indirectly quantify pathogen load. The fully enclosed structure of the system chip minimizes sample and environmental cross-contamination, rendering the assay independent of a specialized laboratory. The portable, fully automated MONITOR facilitates on-site, comprehensive detection of multiple pathogens, even non-specialized operators with minimal training. This presents a promising approach for the rapid and precise diagnosis of influenza-like illness in grassroots communities and underserved rural areas lacking centralized laboratories.

**IMPORTANCE:**

This study combines quantitative polymerase chain reaction (qPCR) and microfluidics to introduce MONITOR, a portable field detection system for multiple pathogens causing influenza-like illness. MONITOR can be rapidly deployed to enable simultaneous sample-in-result-out detection of eight common influenza-like illness (ILI) pathogens with heightened sensitivity and specificity. It is particularly well suited for communities and regions without centralized laboratories, offering robust technical support for the prompt and accurate monitoring and detection of ILI. It holds the potential to be a potent tool in the early detection and prevention of infectious diseases.

## INTRODUCTION

Respiratory infections contribute significantly to global morbidity and mortality annually (1). These pathogens, commonly transmitted via droplets and aerosols, tend to spread rapidly, necessitating vigilant surveillance within global health systems. Initial clinical presentations of these infections often lack specificity, encompassing symptoms like fever, cough, sore throat, nasal congestion, myalgias, and fatigue. Termed influenza-like illness (ILI), these manifestations closely resemble influenza symptoms (2). Monitoring ILI plays a pivotal role in the early identification of epidemic pathogens and sudden outbreaks. This enables precise outbreak risk assessment and the implementation of suitable public health interventions, ultimately curtailing pathogen spread, reducing disease burden, and minimizing severe complications and mortality. Consequently, many countries employ ILI as an indicator to track respiratory infections and provide preemptive alerts regarding emerging infectious diseases (1, 3 – 5). The global COVID-19 pandemic, responsible for widespread devastation, was initially isolated from throat swabs of patients with respiratory infections (6). In 2022, the World Health Organization (WHO) categorized monkeypox virus as a moderate public health concern given its droplet-based transmission potential and human-to-human spread (6). Common pre-rash symptoms in infected individuals comprise fever (62%), drowsiness (41%), myalgias (31%), and headache (27%) (7, 8). A multicenter study in France discovered that influenza viruses and non-influenza respiratory viruses (NIRVs) contributed to influenza-like syndromes in approximately 38% and 15% of patients, respectively, accounting for 5% and 4% of patient deaths (9). Notably, ILI can stem from concurrent or sequential infection with multiple pathogens rather than a single pathogen (10 – 12). A case of co-infection involving monkeypox virus, SARS-CoV-2, and HIV was reported in Italy in July 2022. The patient exhibited prodromal ILI symptoms, including fever, sore throat, malaise, and headache. Monkeypox infection was confirmed 4 days after the patient was diagnosed with SARS-CoV-2 (13). Accurate diagnosis of this ILI arising from multi-pathogen co-infection necessitates more than symptom observation, as reliance on symptoms alone could lead to misdiagnosis, thereby impacting timely and accurate patient management. Prior epidemics, such as COVID-19, underscored the necessity of portable field detection technologies that enable simultaneous detection of multiple ILI-causing pathogens for early diagnosis and public health management (14). Quantitative polymerase chain reaction (qPCR) serves as the gold standard for pathogen identification. Presently, multiplex qPCR is the prevailing method for ILI detection and monitoring. Nonetheless, implementing multiplex PCR demands the incorporation of multiple primer pairs in a single reaction system aimed at specifically amplifying multiple target sites. This approach faces challenges, including primer interference, signal disruption of low-abundance templates by high-abundance templates, and complexities in system optimization. Furthermore, traditional multiplex qPCR requires centralized laboratory resources, costly equipment, intricate sample preparation, and skilled technicians, thereby restricting pathogen testing in grassroots communities and rural areas (15). Microfluidic technology offers several advantages for pathogen detection, encompassing high integration, automation, portability, swift response, and minimal reagent consumption. These attributes render microfluidic technology ideal for creating integrated on-site pathogen detection methodologies, particularly in regions with limited access to central laboratories or restricted pathogen monitoring capabilities. This enhances the ability to address epidemics and public health challenges (16).

In this study, qPCR and microfluidic technology are amalgamated to yield a portable field detection system for various influenza-like illness pathogens, termed the multiplex influenza-like illness microfluidic detection system (MONITOR). This system automates the complete nucleic acid detection process, spanning sample pre-processing to result dissemination. It simultaneously detects eight pathogens, as primers and probes are pre-embedded within parallel, independent chambers. The system prioritizes user-friendliness and efficiency, enabling non-specialists to effortlessly introduce samples for automated detection. MONITOR thus presents a valuable resource for detecting and monitoring influenza-like syndromes in regions with limited medical resources.

## MATERIALS AND METHODS

### Materials and reagents

Reference material of SARS-CoV-2 (2019-nCoV) pseudovirus (Cat. No. GBW09300), monkeypox virus pseudovirus (Cat. No. NIM-RM4059), adenovirus pseudovirus (Cat. No. NIM-RM4065), inactivated influenza B (Victoria) virus (Cat. No. NIM-RM4056), and inactivated influenza A (H1N1) virus (Cat. No. NIM-RM4054) was acquired from the National Institute of Metrology (Beijing, China). Reference material of rhinovirus RNA (Cat. No. NIM-RM4058) was obtained from the Shanghai Institute of Measurement and Testing Technology (Shanghai, China). Reference material of inactivated human metapneumovirus (HMPV) (BDS-IQC-269) and *Mycoplasma pneumoniae* (BDS-IQC-008) was procured from BDS Biological Technology Co., Ltd. (Guangzhou, China). The monkeypox qPCR kit was purchased from Hotgen Biotech Co., Ltd. (Beijing, China). The SARS-CoV-2 qPCR kit was obtained from Sansure Biotech Inc. (Changsha, China). The influenza A virus qPCR kit was sourced from Biogerm Medical Technology Co., Ltd. (Shanghai, China). The influenza B virus qPCR kit was purchased from Ao Dong Inspection and Testing Technology Co., Ltd. (Shenzhen, China). The rhinovirus qPCR kit was acquired from Genmed Gene Medicine Technology Co., Ltd. (Shanghai, China). The human metapneumovirus qPCR kit, *Mycoplasma pneumoniae* qPCR kit, and adenovirus qPCR kit were procured from Huirui Biotechnology Co., Ltd. (Shanghai, China). Artificial saliva (pH 6.8–7) was obtained from Yuanye Biotech (Shanghai, China). Lysis buffer, washing buffer, elution buffer, magnetic beads, PCR buffer, DNA polymerase, reverse transcriptase, dNTPs, magnesium chloride solution, fluorescent probes, primers**,** and quality controls were sourced from Beijing Baicare Biotechnology Co., Ltd. (Beijing, China).

### MONITOR design and operation

**Fig 1:**
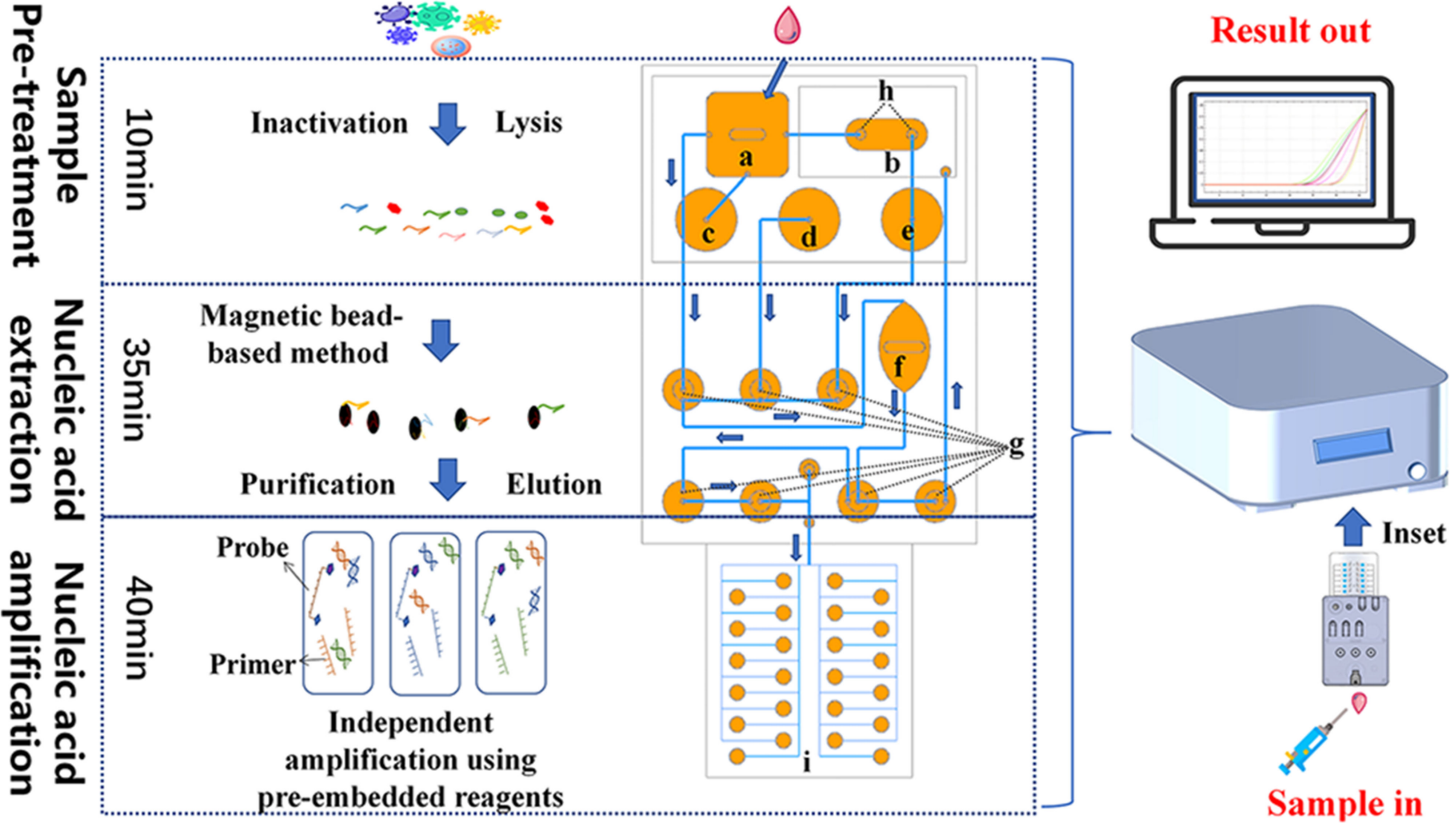
Schematic diagram of MONITOR. MONITOR automates the entire process of multiplex pathogen testing. This is accomplished by employing a sample-in-result-out format and functioning as a modular central laboratory in the field. The microfluidic chip comprises the following components: (a) sample storage chamber, (b) waste fluid retention chamber, (c to e) nucleic acid extraction reagent storage assemblies that store the binding buffer, rinsing buffer, and elution buffer, respectively, (f) nucleic acid extraction cell, (g) fluid isolation valves, and (h) pneumatic valve. Finally, there is the (i) nucleic acid amplification reaction module.

The MONITOR system comprises a microfluidic chip and an Onestart-1000 nucleic acid amplification analyzer. The microfluidic chip integrates microchannels and microvalves to create a microfluidic network, measuring 13.5 cm × 6.5 cm × 2.5 cm. Under pre-programmed control, the pneumatic valve (h) and fluid isolation valve (g) are activated to facilitate fluid flow from the sample storage chamber (a) to the nucleic acid extraction cell (f). Subsequently, the nucleic acid extraction reagent storage assemblies (c–e) employ pressure rods to sequentially introduce the binding buffer (c), rinsing buffer (d), and elution buffer (e) into the nucleic acid extraction cell (f). The magnetic rotor within the nucleic acid extraction cell (f) is activated by the Onestart-1000’s magnetic rotation module, completing nucleic acid purification and elution. Finally, the pre-programmed operation of the pneumatic valve (h) and fluid isolation valve (g) propels the extracted nucleic acids into the nucleic acid amplification reaction module (i), simultaneously expelling waste fluid into the waste fluid retention chamber (b). This process typically occurs within a central laboratory. The MONITOR microfluidic chip features 12 parallel independent chambers, encompassing eight pathogen detection channels, one extraction quality control channel, one amplification quality control channel, and two internal quality control channels. Each chamber includes pre-embedded primers and probes for simultaneous detection of multiple pathogens, with primer details provided in Table S1 in the supplemental material. To initiate the process, the operator combines 300 µL of the sample with 800 µL of lysis buffer and introduces it to the microfluidic chip’s sample inlet. The chip is then placed within the 33 cm × 28 cm × 19 cm Onestart-1000 nucleic acid amplification analyzer, and the amplification protocol is established. The entire detection process can be autonomously executed in as little as 85 minutes (Fig. 1).

### Evaluating the analytical performance of MONITOR

Pathogen standards were employed to assess the analytical performance of MONITOR. To simulate clinical samples, a viral sample fluid containing 1% artificial saliva (ISO/TR10271, pH 6.8–7, Shanghai Yuanye Bio-Technology Co., Ltd.) and 10,000 A549 cells served as the virus dilution matrix. Following FDA guidelines (17), the limit of detection (LoD) was evaluated by diluting the pathogen standards in the virus dilution matrix to concentrations ranging from 0.39 copies/µL to 400 copies/µL and testing each concentration in triplicate. The lowest dilution demonstrating three positive results was deemed the preliminary LoD. The preliminary LoD was tested 20 times, and if over 19 replicates were positive, that dilution was identified as the limit of detection. The analytical specificity of MONITOR was assessed using external quality control samples for the pathogens expected to be detected by MONITOR, encompassing 24 pathogens, including viruses, bacteria, and fungi, as detailed in Table S2.

### Blind study simulated clinical samples

Clinical samples were prepared in a blinded manner by three operators, A, B, and C. Operator A added laboratory-stored pathogen standards and inactivated pathogens to the viral dilution matrix. Operator A prepared 50 samples, encompassing positive samples, mixed samples containing two or more pathogens, and negative samples in duplicate. Operator B conducted the qPCR assay on the 50 samples, while Operator C performed the MONITOR assay on the same samples. Operator B followed the qPCR kit instructions and extracted nucleic acids from the pathogens using a fully automated nucleic acid extractor (Thermo Scientific KingFisher Flex) and associated reagents. Real-time PCR analysis was conducted on a CFX96 touchscreen real-time PCR detection system (Bio-Rad). The duration of the single pathogen assay for a single sample by Operator B was approximately 2 hours 45 minutes. Operators B and C were blinded to the sample contents during the experiment.

### Data analysis

The specificity, sensitivity, and accuracy of the MONITOR diagnostic test were calculated using the following formulas: specificity = TP/(TP + FN) × 100%; sensitivity = TN/(TN + FP) × 100%; and accuracy = (TP + TN)/(TP + TN + FP + FN), where TN, FN, TP, and FP represent true negative, false negative, true positive, and false positive, respectively (18). The kappa test was utilized to assess the consistency of qualitative detection of clinical samples between MONITOR and qPCR. The interpretation of the kappa statistic is as follows: less than 0 indicates poor; 0–0.20, slight; 0.21–0.40, fair; 0.41–0.60, moderate; 0.61–0.80, substantial; and 0.81–1.00, almost perfect consistency (19). Linear regression analysis and Bland-Altman analysis were used to compare cycle threshold (Ct) values of positive samples between MONITOR and qPCR.

## RESULTS

### Limit of detection, linearity, and cross-reactivity

**Fig 2:**
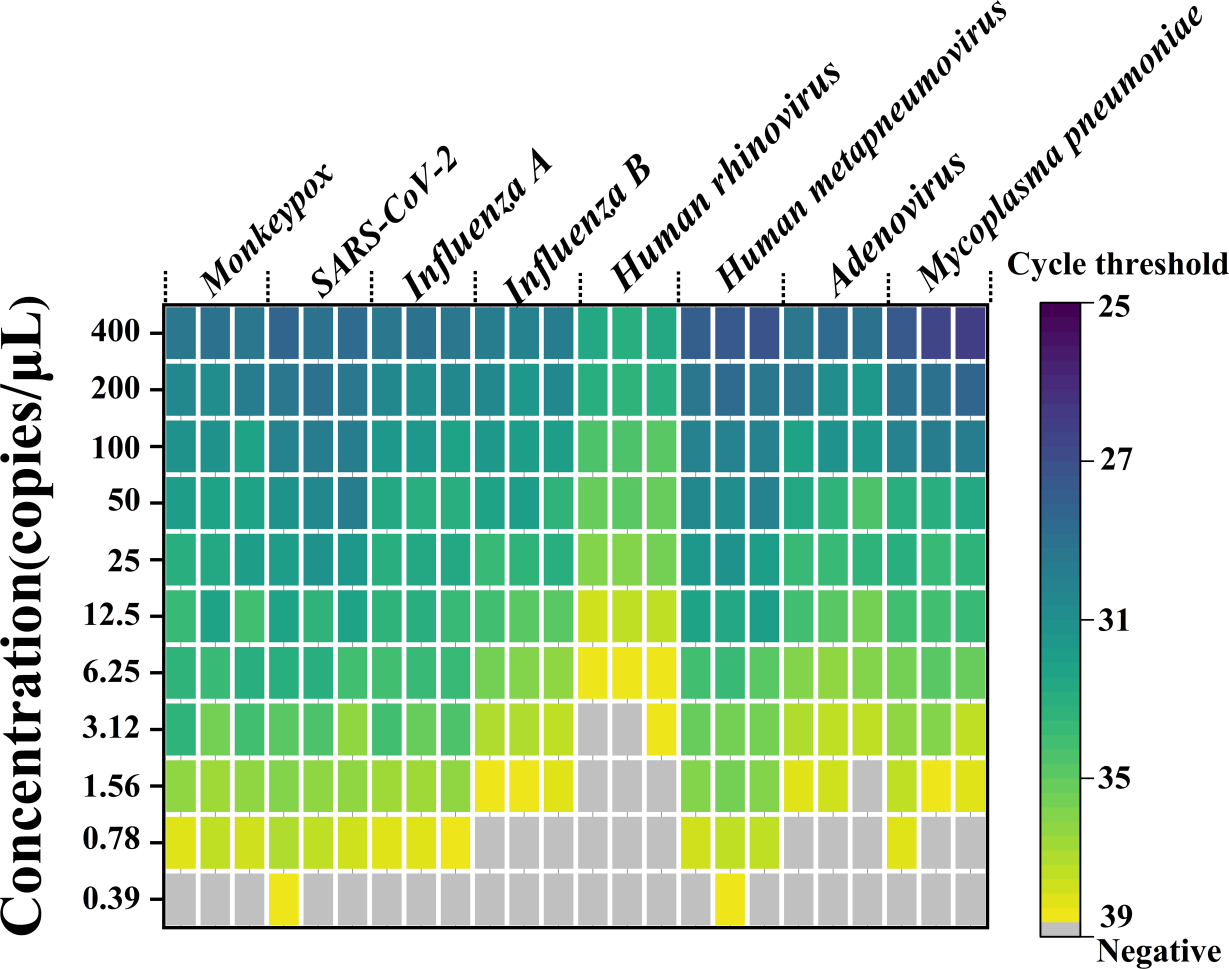
This heatmap illustrates the detection outcomes for eight pathogens using MONITOR. Each concentration underwent testing in triplicate, with the cycle threshold (Ct) values recorded. Ct values exceeding 40 were classified as negative.

In Fig. 2, we present the initial detection limits of MONITOR for various pathogens, such as monkeypox, SARS-CoV-2, influenza A, influenza B, human rhinovirus, human metapneumovirus, adenovirus, and *Mycoplasma pneumoniae.* The detection limits were as follows: 0.78 copies/µL for monkeypox, SARS-CoV-2, and influenza A; 1.56 copies/µL for *Mycoplasma pneumoniae* and influenza B; 6.25 copies/µL for human rhinovirus; and 3.12 copies/µL for adenovirus.

To assess the LoD of the MONITOR system, we performed 20 preliminary LoD tests for each pathogen. The results are summarized in Table 1. The relative standard deviation (RSD) of the 20 repeated tests for the preliminary LoD by MONITOR was consistently below 5%, indicating robust precision. Following the criterion of an RSD of <25% for defining the limit of quantification (LoQ) and considering a minimum sample volume of 300 µL for clinical samples with MONITOR, the LoQs were as follows: 234 copies/reaction for monkeypox, SARS-CoV-2, influenza A, and influenza B; 1,875 copies/reaction for human rhinovirus; 936 copies/reaction for human metapneumovirus; 468 copies/reaction for adenovirus; and 468 copies/reaction for *Mycoplasma pneumoniae*. These LoQs fulfill clinical detection requirements (Table 1) (20 – 22).

**TABLE 1:** The LoDs and LoQs of MONITOR

Pathogen species	LoD (copies/µL)	LoQ (copies/reaction)	No. of positives/no. of testing replicates
Monkeypox	0.78	234	20/20
SARS-CoV-2	0.78	234	20/20
Influenza A	0.78	234	20/20
Influenza B	1.56	468	19/20
Rhinovirus	6.25	1875	19/20
HMPV	0.78	234	20/20
Adenovirus	3.12	936	20/20
*Mycoplasma pneumoniae*	1.56	468	19/20

**Fig 3:**
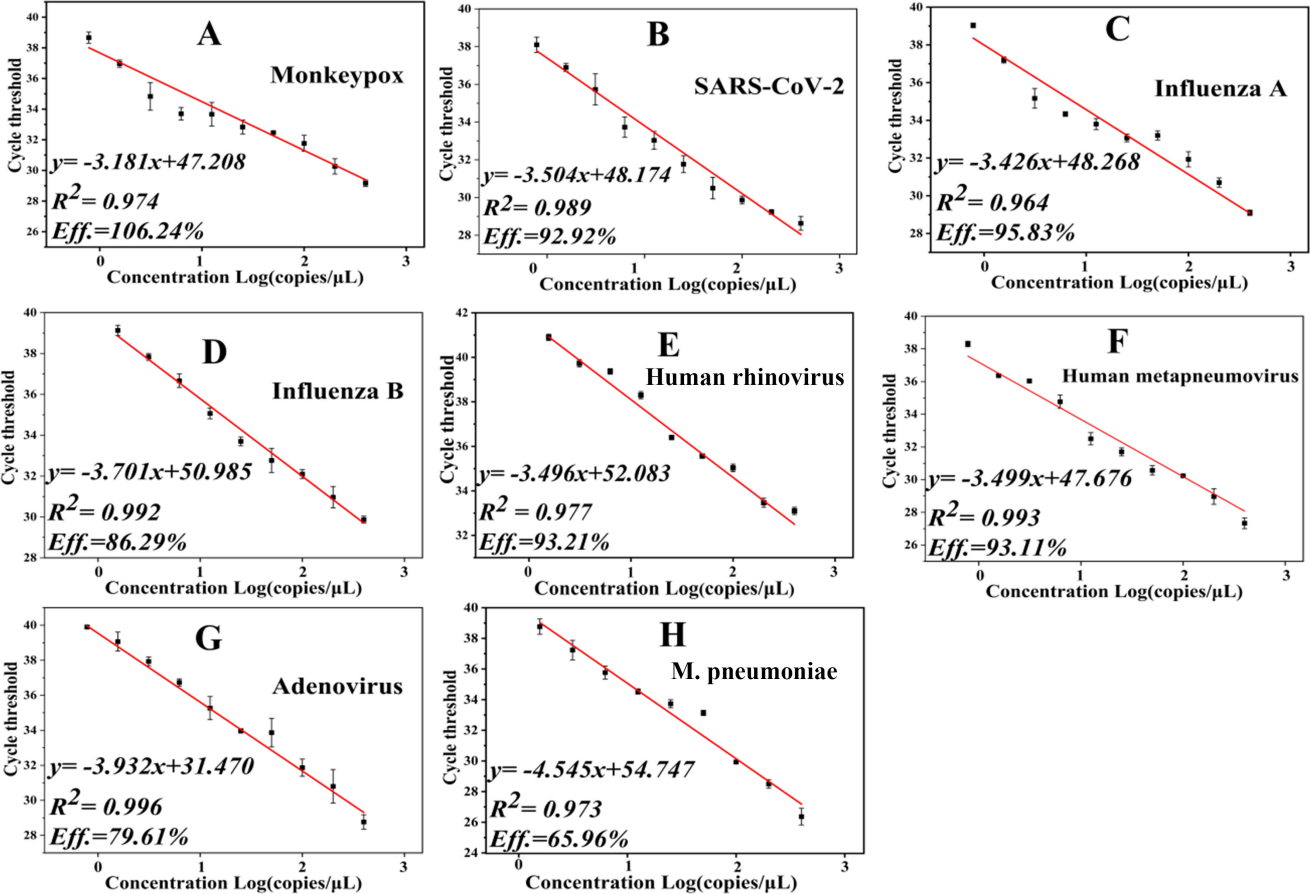
Assessment of MONITOR’s detection performance using pathogen standard products. The standard curves for the eight pathogens tested by MONITOR are illustrated as follows: (A) monkeypox, (B) SARS-CoV-2, (C) influenza A, (D) influenza B, (E) human rhinovirus, (F) human metapneumovirus, (G) adenovirus, and (H) *Mycoplasma pneumoniae*. The concentration range of the tested standard products ranged from 0.39 copies/µL to 400 copies/µL. The error bars depict the standard deviation of the average value from three replicates.

The MONITOR system’s real-time PCR standard curves were generated by plotting Ct values against viral load using pseudovirus or inactivated virus reference materials. All eight standard curves exhibited satisfactory linearity and repeatability. The Ct value RSD for various concentrations of the eight pathogens was consistently below 5%. The linear correlation coefficients (*R*²) of the standard curves exceeded 0.964. MONITOR showed relatively high amplification efficiencies (Eff.) ranging from 92.92% to 106.24% for monkeypox, SARS-CoV-2, influenza A, human rhinovirus, and human metapneumovirus. In contrast, amplification efficiencies for influenza B and adenovirus were 86.29% and 79.61%, respectively. Furthermore, *Mycoplasma pneumoniae* demonstrated a lower amplification efficiency of 65.91% (Fig. 3).

### Specificity detection

**Fig 4:**
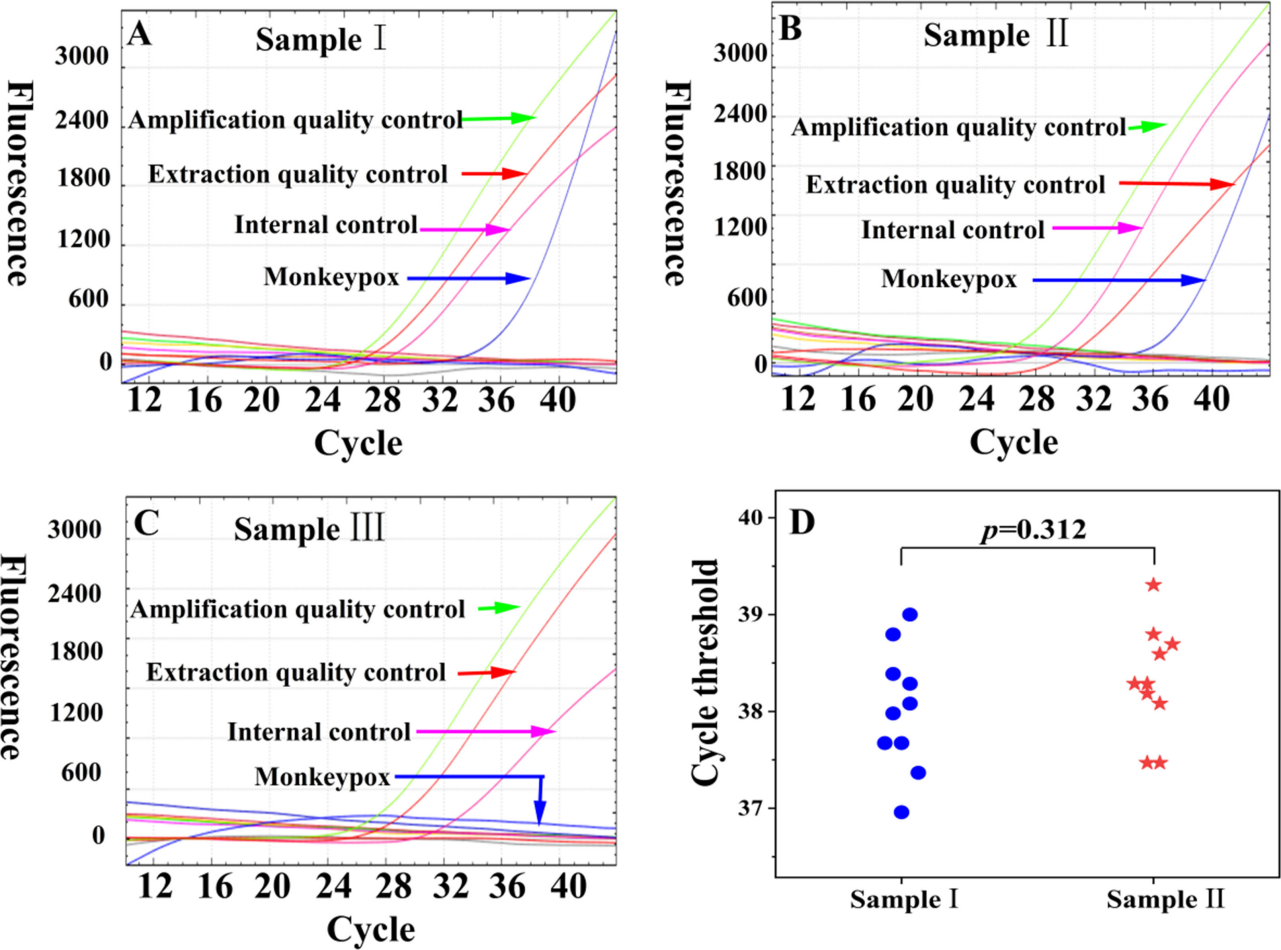
Specificity of MONITOR for monkeypox. To verify the specificity of MONITOR for monkeypox, three clinically simulated samples were prepared: sample I (monkeypox sample) containing 0.78 copies/µL of monkeypox reference material; sample II (mixed orthopoxvirus mixed sample) containing 0.78 copies/µL of monkeypox virus reference material, 100 copies/µL of vaccine virus, and 100 copies/µL of cowpox virus; and sample III (orthopoxvirus mixed samples) containing 100 copies/µL of vaccine virus and 100 copies/µL of cowpox virus. Amplification curves of MONITOR detection for samples I, II, and III are displayed in panels A to C, respectively. Panel D illustrates the cycle threshold of MONITOR repeated detection for 10 times in samples I and II.

We assessed MONITOR’s specificity by detecting nucleic acid control materials from 24 distinct viruses, bacteria, and fungi. The results indicated that MONITOR exhibited no false positives for any of the 24 tested pathogens, signifying excellent specificity. Refer to Table S2 for the list of pathogens. It is noteworthy that previous studies identified cross-reactivity of certain commercially available monkeypox qPCR kits with vaccinia virus and cowpox virus (23). MONITOR successfully detected 0.78 copies/µL of monkeypox virus standard material when mixed with highly concentrated vaccine virus and cowpox virus quality control samples. No non-specific amplification curves were observed for the vaccine virus and cowpox virus. MONITOR showed no statistically significant difference in the cycle threshold for the 0.78 copies/µL monkeypox standard material and the mixed sample containing monkeypox, vaccine virus, and cowpox virus (Fig. 4D), affirming its specificity against interference.

### MONITOR detection performance for simulated clinical samples

**Fig 5:**
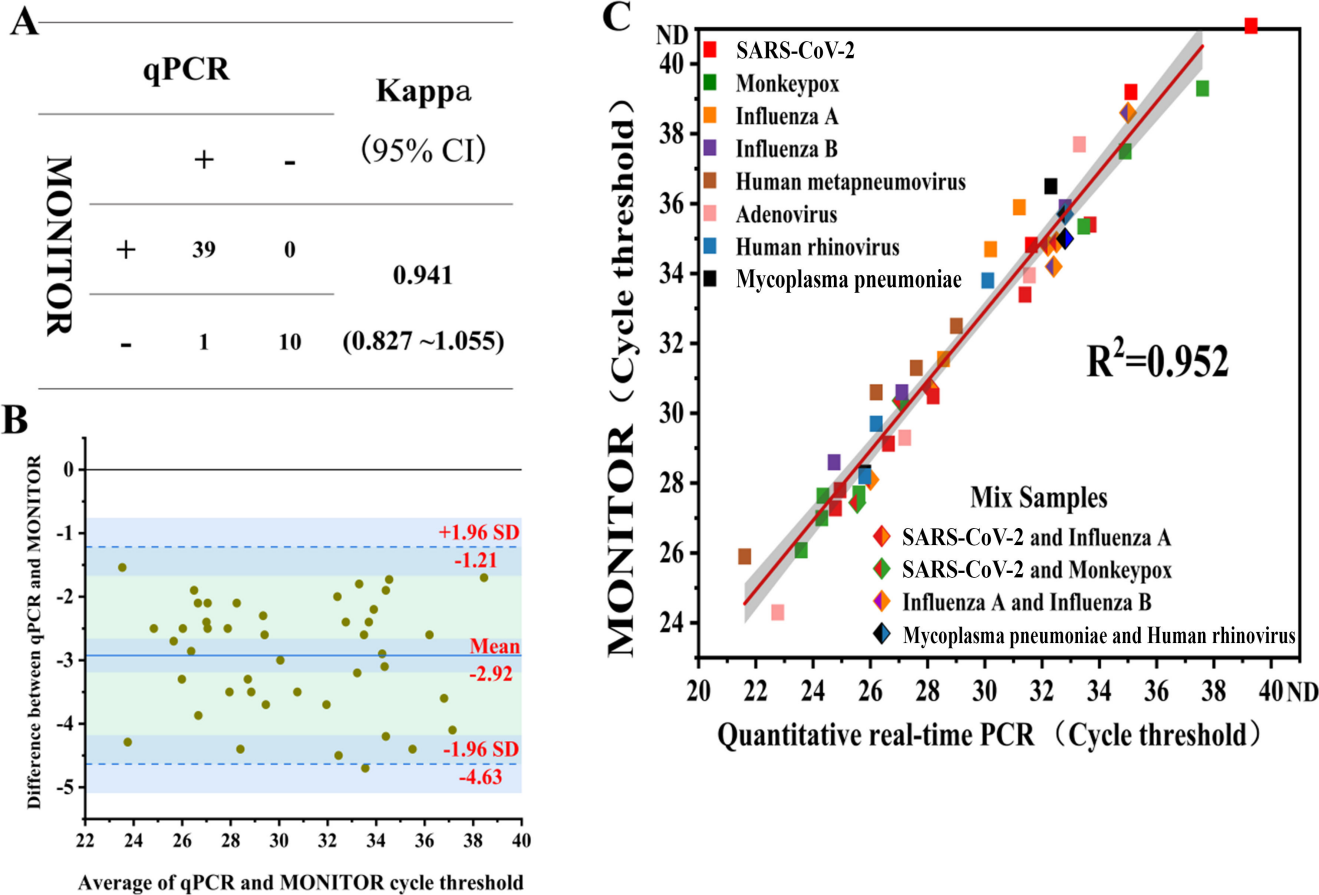
Evaluation of MONITOR detection performance for simulated clinical samples compared with qPCR. (**A**) Concordance between MONITOR and qPCR for 50 simulated clinical samples. (**B**) Bland-Altman analysis of positive simulated clinical samples. (**C**) Linear regression combined with a 95% confidence interval analysis of the correlation between MONITOR and qPCR cycle thresholds for positive samples. ND, not detected.

Figure 5A illustrates the results of qualitative testing of 50 simulated clinical samples using MONITOR and qPCR. These samples comprised 10 negative samples and 5 mixed samples, including 2 mixed samples of SARS-CoV-2 with influenza A, 1 mixed sample of influenza A with influenza B, 1 mixed sample of monkeypox with SARS-CoV-2, and 1 mixed sample of rhinovirus with *Mycoplasma pneumoniae*. Both qPCR and MONITOR accurately identified all negative and positive samples, except for one low viral load SARS-CoV-2 sample that was falsely negative by MONITOR. The kappa concordance test showed an almost perfect concordance between the two methods, with a kappa coefficient of 0.941 (95% CI 0.827–1.055). The mean difference between MONITOR and qPCR cycle threshold values was −2.92, with upper and lower 95% confidence intervals for the cycle threshold difference at −1.21 and −4.63, respectively. Except for one influenza A simulated clinical sample exceeding the lower 95% confidence interval, all positive simulated samples fell within the 95% confidence interval. This signifies strong agreement between the cycle threshold values of the two methods. Linear regression analysis of MONITOR and qPCR cycle thresholds for positive samples revealed a high correlation coefficient (*R*² value of 0.952, *P* < 0.001), indicating that MONITOR-derived cycle threshold values can indirectly characterize pathogen load in the samples. A comparative study of three multiplex platforms for respiratory pathogens indicates that the leading multiplex detection platforms on the market, including the BioFire FilmArray Respiratory Panel (BioFire Diagnostics, Salt Lake City, UT), the Luminex NxTag Respiratory Pathogen Panel (Luminex Corporation, Austin, TX), and the TaqMan Array Card (Life Technologies, Carlsbad, CA), have an overall concordance of approximately 97% with the gold standard RT-PCR. In the case of samples with Ct > 35, the false negative rate is approximately 50% (24). The performance of the MONITOR system is essentially equivalent to the three multiplex detection platforms mentioned above.

## DISCUSSION

Given the widespread prevalence of pathogens like the influenza virus, SARS-CoV-2, and monkeypox virus that can trigger non-specific ILI symptoms, it becomes crucial to swiftly and accurately monitor ILI pathogens for effective public health management and epidemic control. In our study, we successfully developed a portable and automated microfluidic detection system for multiple influenza-like syndrome pathogens.

MONITOR combines microfluidic chip technology and PCR technology to achieve highly sensitive and specific yet simple and automated detection of multiple pathogens. The system’s detection limit for the eight target pathogens, determined using FDA guidelines, ranged from 0.78 to 6.25 copies/µL, thus meeting clinical detection requirements. The standard curves of MONITOR for these eight pathogens displayed favorable linearity and repeatability. The cycle threshold RSD of MONITOR for various concentrations of the target pathogens remained under 5%, with a linear correlation coefficient (*R*²) surpassing 0.964 and a high amplification efficiency, underscoring its consistent and sensitive detection capabilities. Evaluation using blinded, simulated clinical samples showcased MONITOR’s sensitivity of 97.5%, specificity of 100%, and accuracy of 98% (Fig. S1). Moreover, the cycle threshold of the target viruses in MONITOR exhibited robust consistency with measurements obtained via qPCR, demonstrating its indirect quantitative potential for assessing target pathogens in samples.

In practical on-site applications, MONITOR presents distinct advantages over multiplex qPCR. With an integrated detection chip, MONITOR simplifies operations. Operators merely need to add the sample to the microfluidic chip and then insert it into the device, a process completed in less than a minute. Within 85 minutes, the system autonomously completes the “sample-in-result-out” procedure. Moreover, MONITOR’s fully enclosed system chip design substantially minimizes the risks of sample and environmental cross-contamination. In contrast to traditional multiplex qPCR, which demands centralized laboratory facilities, costly equipment, intricate sample preparation, and skilled personnel, MONITOR can be promptly deployed on-site by an individual to detect a range of ILI-related pathogens. The streamlined testing process minimizes the potential for human operational errors, enhancing the reliability and stability of detection. During public health emergencies, MONITOR’s automation, portability, and one-stop operation can significantly expedite detection and furnish accurate data to guide control measures. Additionally, it offers a promising avenue for the rapid and precise diagnosis and monitoring of influenza-like syndromes in grassroots communities and rural areas without centralized detection facilities.

However, it is important to note that while MONITOR exhibited excellent performance in our study, its efficacy in real clinical and public health scenarios necessitates further validation through larger sample sizes and multicenter studies. Furthermore, although MONITOR can detect eight common ILI pathogens, it does not encompass all potential pathogens. Therefore, to broaden its response to diverse public health emergencies, optimizing and expanding MONITOR’s detection scope to encompass newly discovered and region-specific pathogens becomes essential. The design of multiple parallel and independent assay chambers in MONITOR helps mitigate the interference issue posed by various target primers encountered in traditional multiplex qPCR. This design empowers MONITOR to utilize primers and probes validated by standard PCR without necessitating extra optimization. This adaptability allows for tailoring the detection spectrum as per application requirements, facilitating swift responses to varying public health emergencies.

In conclusion, MONITOR can be swiftly deployed in the realm of infectious diseases, enabling the simultaneous detection of eight common ILI pathogens with high sensitivity and specificity, leading to a seamless sample-in-result-out process. It holds particular promise for communities and regions lacking centralized laboratories, offering valuable technical assistance for rapid and precise ILI monitoring and detection. Its potential as a potent tool for the early detection and prevention of infectious diseases is considerable.

## SUPPLEMENTAL MATERIAL

Supplemental material spectrum.02320-23-s0001.docx Tables S1 and S2; Fig. S1.

ASM does not own the copyrights to Supplemental Material that may be linked to, or accessed through, an article. The authors have granted ASM a non-exclusive, world-wide license to publish the Supplemental Material files. Please contact the corresponding author directly for reuse.

## References

[1] Iuliano AD , Roguski KM , Chang HH , Muscatello DJ , Palekar R , Tempia S , Cohen C , Gran JM , Schanzer D , Cowling BJ , Wu P , Kyncl J , Ang LW , Park M , Redlberger-Fritz M , Yu H , Espenhain L , Krishnan A , Emukule G , van Asten L , Pereira da Silva S , Aungkulanon S , Buchholz U , Widdowson M-A , Bresee JS , Global Seasonal Influenza-associated Mortality Collaborator Network . 2018. Estimates of global seasonal influenza-associated respiratory mortality: a modelling study. Lancet 391:1285–1300. doi:10.1016/S0140-6736(17)33293-2 29248255 PMC5935243

[2] Gandhi L , Maisnam D , Rathore D , Chauhan P , Bonagiri A , Venkataramana M . 2022. Respiratory illness virus infections with special emphasis on COVID-19. Eur J Med Res 27:236. doi:10.1186/s40001-022-00874-x 36348452 PMC9641310

[3] Osthus D , Moran KR . 2021. Multiscale influenza forecasting. Nat Commun 12:2991. doi:10.1038/s41467-021-23234-5 34016992 PMC8137955

[4] Li JH , Wu CC , Tseng YJ , Han ST , Pekosz A , Rothman R , Chen KF . 2023. Applying symptom dynamics to accurately predict influenza virus infection: an international multicenter influenza-like illness surveillance study. Influenza Other Respir Viruses 17:e13081. doi:10.1111/irv.13081 36480419 PMC9835452

[5] Huang W-J , Cheng Y-H , Tan M-J , Liu J , Li X-Y , Zeng X-X , Tang J , Wei H-J , Chen T , Yang L , Xie Y-R , Yang J-Y , Xiao N , Wang D-Y . 2022. Epidemiological and virological surveillance of influenza viruses in China during 2020–2021. Infect Dis Poverty 11:74. doi:10.1186/s40249-022-01002-x 35768826 PMC9244124

[6] Huang C , Wang Y , Li X , Ren L , Zhao J , Hu Y , Zhang L , Fan G , Xu J , Gu X , Cheng Z , Yu T , Xia J , Wei Y , Wu W , Xie X , Yin W , Li H , Liu M , Xiao Y , Gao H , Guo L , Xie J , Wang G , Jiang R , Gao Z , Jin Q , Wang J , Cao B . 2020. Clinical features of patients infected with 2019 novel coronavirus in Wuhan, China. Lancet 395:497–506. doi:10.1016/S0140-6736(20)30183-5 31986264 PMC7159299

[7] Thornhill JP , Barkati S , Walmsley S , Rockstroh J , Antinori A , Harrison LB , Palich R , Nori A , Reeves I , Habibi MS , Apea V , Boesecke C , Vandekerckhove L , Yakubovsky M , Sendagorta E , Blanco JL , Florence E , Moschese D , Maltez FM , Goorhuis A , Pourcher V , Migaud P , Noe S , Pintado C , Maggi F , Hansen A-BE , Hoffmann C , Lezama JI , Mussini C , Cattelan A , Makofane K , Tan D , Nozza S , Nemeth J , Klein MB , Orkin CM , SHARE-net Clinical Group . 2022. Monkeypox virus infection in humans across 16 countries - April-June 2022. N Engl J Med 387:679–691. doi:10.1056/NEJMoa2207323 35866746

[8] Kozlov M . 2022. How does monkeypox spread? What scientists know. Nature 608:655–656. doi:10.1038/d41586-022-02178-w 35953575

[9] Bénézit F , Loubet P , Galtier F , Pronier C , Lenzi N , Lesieur Z , Jouneau S , Lagathu G , L’Honneur A-S , Foulongne V , Vallejo C , Alain S , Duval X , Houhou N , Costa Y , Vanhems P , Amour S , Carrat F , Lina B , Launay O , Tattevin P , FLUVAC Study Group . 2020. Non-influenza respiratory viruses in adult patients admitted with influenza-like illness: a 3-year prospective multicenter study. Infection 48:489–495. doi:10.1007/s15010-019-01388-1 32056143 PMC7095392

[10] Ceccarelli G , d’Ettorre G , Russo A , Fabris S , Ciccozzi M , d’Ettorre G . 2023. SARS-CoV-2 pandemic, influenza, and influenza-like illness epidemics allies or enemies? J Med Virol 95:e28148. doi:10.1002/jmv.28148 36114792 PMC9538197

[11] Piret J , Boivin G . 2022. Viral interference between respiratory viruses. Emerg Infect Dis 28:273–281. doi:10.3201/eid2802.211727 35075991 PMC8798701

[12] Swets MC , Russell CD , Harrison EM , Docherty AB , Lone N , Girvan M , Hardwick HE , ISARIC4C Investigators, Visser LG , Openshaw PJM , Groeneveld GH , Semple MG , Baillie JK . 2022. SARS-CoV-2 co-infection with influenza viruses, respiratory syncytial virus, or adenoviruses. Lancet 399:1463–1464. doi:10.1016/S0140-6736(22)00383-X 35344735 PMC8956294

[13] Nolasco S , Vitale F , Geremia A , Tramuto F , Maida CM , Sciuto A , Coco C , Manuele R , Frasca E , Frasca M , Magliocco S , Gennaro A , Tumino E , Maresca M , Montineri A . 2023. First case of monkeypox virus, SARS-CoV-2 and HIV co-infection. J Infect 86:e21–e23. doi:10.1016/j.jinf.2022.08.014 35995308 PMC9389837

[14] Gupta S , Gupta T , Gupta N . 2022. Global respiratory virus surveillance: strengths, gaps, and way forward. Int J Infect Dis 121:184–189. doi:10.1016/j.ijid.2022.05.032 35584744 PMC9107382

[15] Zhang N , Wang L , Deng X , Liang R , Su M , He C , Hu L , Su Y , Ren J , Yu F , Du L , Jiang S . 2020. Recent advances in the detection of respiratory virus infection in humans. J Med Virol 92:408–417. doi:10.1002/jmv.25674 31944312 PMC7166954

[16] Kumar A , Parihar A , Panda U , Parihar DS . 2022. Microfluidics-based point-of-care testing (POCT) devices in dealing with waves of COVID-19 pandemic: The emerging solution. ACS Appl Bio Mater 5:2046–2068. doi:10.1021/acsabm.1c01320 35473316

[17] U. S Food & Drug Administration . In vitro diagnostics EUAs. https://www.fda.gov/medical-devices/coronavirus-disease-2019-covid-19-emergency-use-authorizations-medical-devices/vitro-diagnostics-euas.

[18] Huang H , Huang K , Sun Y , Luo D , Wang M , Chen T , Li M , Duan J , Huang L , Dong C . 2022. A Digital Microfluidic RT-qPCR platform for multiple detections of respiratory pathogens. Micromachines (Basel) 13:1650. doi:10.3390/mi13101650 36296002 PMC9611846

[19] Liu J , Kabir F , Manneh J , Lertsethtakarn P , Begum S , Gratz J , Becker SM , Operario DJ , Taniuchi M , Janaki L , Platts-Mills JA , Haverstick DM , Kabir M , Sobuz SU , Nakjarung K , Sakpaisal P , Silapong S , Bodhidatta L , Qureshi S , Kalam A , Saidi Q , Swai N , Mujaga B , Maro A , Kwambana B , Dione M , Antonio M , Kibiki G , Mason CJ , Haque R , Iqbal N , Zaidi AKM , Houpt ER . 2014. Development and assessment of molecular diagnostic tests for 15 enteropathogens causing childhood diarrhoea: a multicentre study. Lancet Infect Dis 14:716–724. doi:10.1016/S1473-3099(14)70808-4 25022434

[20] Kralik P , Ricchi M . 2017. A basic guide to real time PCR in microbial diagnostics: definitions, parameters, and everything. Front Microbiol 8:108. doi:10.3389/fmicb.2017.00108 28210243 PMC5288344

[21] Viloria Winnett A , Akana R , Shelby N , Davich H , Caldera S , Yamada T , Reyna JRB , Romano AE , Carter AM , Kim MK , Thomson M , Tognazzini C , Feaster M , Goh YY , Chew YC , Ismagilov RF . 2023. Extreme differences in SARS-CoV-2 viral loads among respiratory specimen types during presumed pre-infectious and infectious periods. PNAS Nexus 2:pgad033. doi:10.1093/pnasnexus/pgad033 36926220 PMC10013338

[22] Feikin DR , Fu W , Park DE , Shi Q , Higdon MM , Baggett HC , Brooks WA , Deloria Knoll M , Hammitt LL , Howie SRC , Kotloff KL , Levine OS , Madhi SA , Scott JAG , Thea DM , Adrian PV , Antonio M , Awori JO , Baillie VL , DeLuca AN , Driscoll AJ , Ebruke BE , Goswami D , Karron RA , Li M , Morpeth SC , Mwaba J , Mwansa J , Prosperi C , Sawatwong P , Sow SO , Tapia MD , Whistler T , Zaman K , Zeger SL , O’ Brien KL , Murdoch DR , PERCH Study Group . 2017. Is higher viral load in the upper respiratory tract associated with severe pneumonia? findings from the PERCH study. Clin Infect Dis 64:S337–S346. doi:10.1093/cid/cix148 28575373 PMC5447843

[23] Michel J , Targosz A , Rinner T , Bourquain D , Brinkmann A , Sacks JA , Schaade L , Nitsche A . 2022. Evaluation of 11 commercially available PCR kits for the detection of monkeypox virus DNA Berlin, July to September 2022. Euro Surveill 27:2200816. doi:10.2807/1560-7917.ES.2022.27.45.2200816 36367010 PMC9650706

[24] Banerjee D , Hassan F , Avadhanula V , Piedra PA , Boom J , Sahni LC , Weinberg GA , Lindstrom S , Rha B , Harrison CJ , Selvarangan R . 2022. Comparative analysis of three multiplex platforms for the detection of respiratory viral pathogens. J Clin Virol 156:105274. doi:10.1016/j.jcv.2022.105274 36099751

