# Supplementary material for: Multiplexed on-site sample-in-result-out test through microfluidic real-time PCR (MONITOR) for the detection of multiple pathogens causing influenza-like illness
Source: Microbiol Spectr. 2023 Oct 27;11(6):e02320-23. doi: 10.1128/spectrum.02320-23 (PMC10714808; doi:10.1128/spectrum.02320-23)
Supplement: Supplemental material — Tables S1 and S2; Fig. S1. [file spectrum.02320-23-s0001.docx]

Supplementary information to

**Multiplexed On-site sample-in-result-out Test through Microfluidic Real-time PCR（MONITOR）of Detection Multiple Pathogens Causing Influenza-Like Illness**

Yi Yang^†a^, Chao Wang^†a^, Hua Shi^†a^, Xudong Guo^a^, Wanying Liu ^a^, Jinhui Li ^a^, Lizhong Li^a^, Jun Zhao ^a^, Guohao Zhang^b^, Hongbin Song^*a^, Rongzhang Hao^*c^, Rongtao Zhao^*a^

a. Chinese PLA Center for Disease Control and Prevention, Beijing, 100071, China

b. Beijing Baicare Biotechnology Co., Ltd., Beijing 102206, China

c. Department of Toxicology and Sanitary Chemistry, School of Public Health, Capital Medical University, Beijing, 100069, China.

^†^These authors have equal contribution to this work.

***Corresponding author**. Email address: zhaorongtao1984@163.com (R. Zhao), hao@ccmu.edu.cn (R. Hao), hongbinsong@263.net（H. Song）

**Supplementary file 1:**

Table S1 List of MONITOR primers and probes synthesized in this study.

| Channel | Targets | sequences (5′→3′) | Gene target |
| --- | --- | --- | --- |
| 1 | *Monkeypox* | F: CGGTGGCGTTATACATCTTA | *B6R* |
|  |  | R:CGTACACATGTTGGGAGTAT |  |
|  |  | P:FAM-ACACTAACGGGGTCTCCATCATCCACA-BHQ1 |  |
| *2* | *SARS-COV-2* | F:CCCTGTGGGTTTTACACTTAA | *ORF1ab* |
|  |  | R:ACGATTGTGCATCAGCTGA |  |
|  |  | P:FAM-CCGTCTGCGGTATGTGGAAAGGTTATGG-BHQ1 |  |
| *3* | *Influenza A* | F:GAGTGGCTAAAGACAAGA | *MP* |
|  |  | R:TCTCCATTYCCATTDAGR |  |
|  |  | P:FAM-ACCGTGCCCAGTGA-BHQ1 |  |
| *4* | *Influenza B* | F:TCCTCAACTCACTCTTCGAGCG | *N* |
|  |  | R:CGGTGCTCTTGACCAAATTGG |  |
|  |  | P:FAM-CCAATTCGAGCAGCTGAAACTGCGGTG-BHQ1 |  |
| *5* | *Rhinovieus* | F:CTTCTRTCATCCAGCAAATAYACC | *5’UTR* |
|  |  | R:ACCCAGTRAATTTATGATTAGCATC |  |
|  |  | P:FAM-TCAATRCTRTCTCCTGTGCTCCGTTG-BHQ1 |  |
| *6* | *HMPV* | F:CATCAGGYAAYATYCCACAAAA | *N* |
|  |  | R:AGGGCACCTACACAYAATAARATT |  |
|  |  | P:FAM-AGAGRCCTTCAGCACCAGACACACCWA-BHQ1 |  |
| *7* | *Adenovirus* | F:GCCACGGTGGGGTTTCTAAACTT | *Hexon* |
|  |  | R:GCCCCAGTGGTCTTACATGCACATC |  |
|  |  | P:FAM-TGCACCAGACCCGGGCTCAGGTACTCCGA-BHQ1 |  |
| *8* | *Mycoplasma.Pneumoniue* | F:CCACRCCAATGCCATCA | *P1* |
|  |  | R:GGASGAAAAGCTCGTGTTACGA |  |
|  |  | P:FAM-CCGCGCTTAACCCCGTGAACG-BHQ1 |  |

**Supplementary file 2:**

Table S2 The specificity performance of MONITOR has been the subject of testing. MONITOR was used to test for viruses, bacteria and fungi on a range of external quality control samples. No false positive results were obtained.

| **Pathogen** | **Source** | **No.test** | **Result** |
| --- | --- | --- | --- |
| Vaccinia virus | Shanghai Zhaorui Biological Technology Co.,Ltd. | 3 | Negative |
| [Cowpox virus](https://www.baidu.com/link?url=XHBaWCdCRpIGSmN4kZg1yJkkKbDjeo5b_h4P65X2TnWUtlMD3EhF-0uQA_CTmIaO2SscZel7QrShsn4e5fb4vVgLiF-RzbbmH60HZyr3pZDn51smHHt9A8MxwIHk6zps&wd=&eqid=8570a3ab0002084b000000036447c452) |  | 3 | Negative |
| Staphylococcus aureus | China General Microbiological Culture Collection Center | 3 | Negative |
| Klebsiella pneumoniae |  | 3 | Negative |
| Moraxella catarrhalis | Guangdong Microbial Culture Collection Center | 3 | Negative |
| Legionella pneumophila |  | 3 | Negative |
| Haemophilus influenzae |  | 3 | Negative |
| Staphylococcus epidermidis |  | 3 | Negative |
| Candida albicans | China national institutes for food and drug control | 3 | Negative |
| Escherichia Coli |  | 3 | Negative |
| Streptococcus pneumoniae |  | 3 | Negative |
| Acinetobacter baumannii | [China national Research Institute of Food & Fermentation Industries Co., Ltd.](http://www.cnif.cn/cnifen/5042.html) | 3 | Negative |
| Herpes simplex virus 1 | Guangzhou BDS Biological Technology Co., Ltd | 3 | Negative |
| Human cytomegalovirus |  | 3 | Negative |
| Epstein-barr virus |  | 3 | Negative |
| Chlamydia pneumoniae |  | 3 | Negative |
| Coronavirus 229E |  | 3 | Negative |
| Coronavirus OC43 type |  | 3 | Negative |
| Coronavirus NL63 type | Guangzhou BDS Biological Technology Co., Ltd | 3 | Negative |
| Coronavirus HKU1 type |  | 3 | Negative |
| Parainfluenza virus type 1 |  | 3 | Negative |
| Parainfluenza virus type 3 |  | 3 | Negative |
| Corynebacterium genus | [Beijing BeNa Culture Collection Technology Co., Ltd](https://www.bncc.com/) | 3 | Negative |
| Lactobacillus acidophilus |  | 3 | Negative |

**Supplementary file 3:**

**
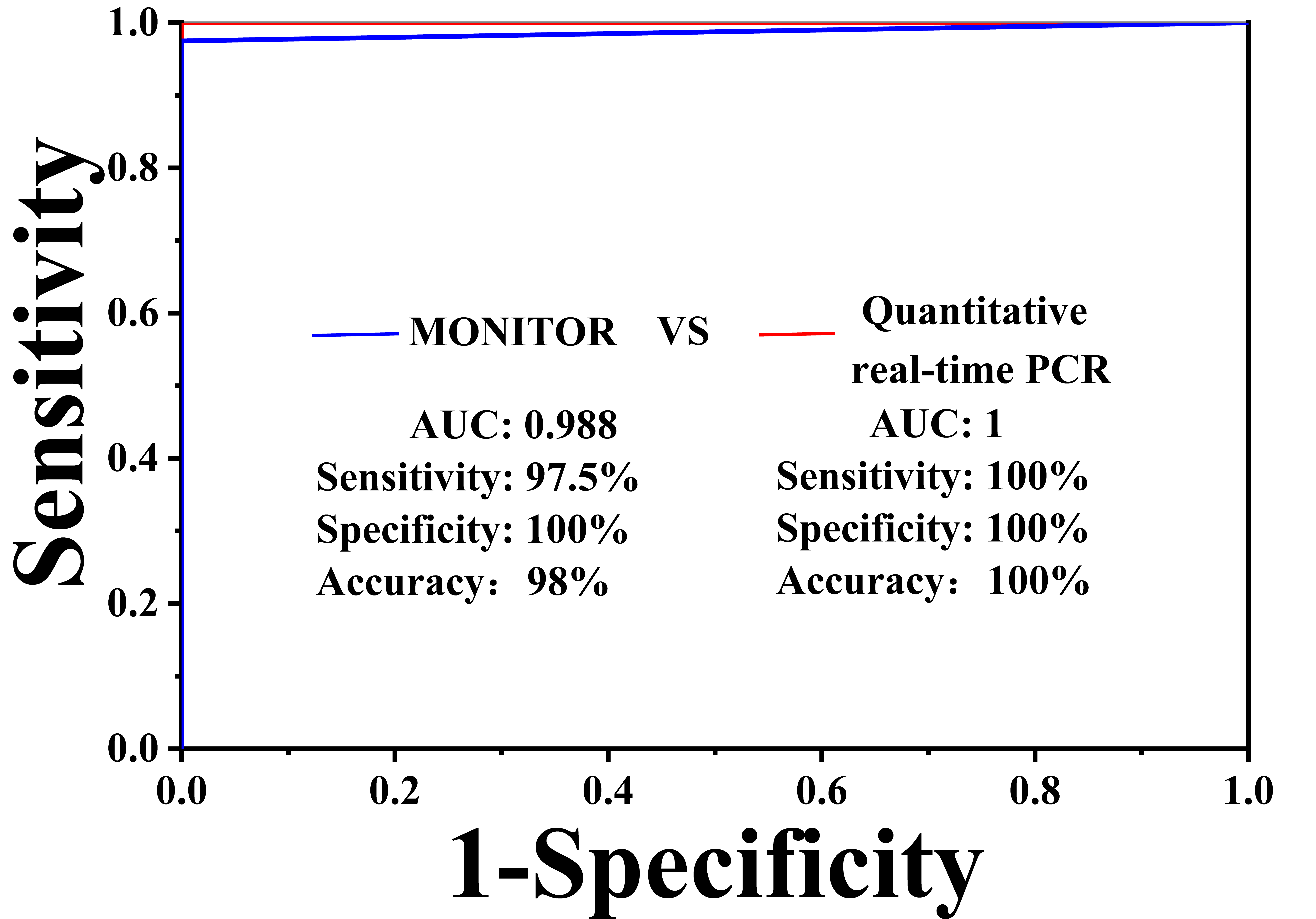
**

Figure S1 ROC curve for detecting clinical samples by the MONITOR and quantitative real-time PCR .
